# Supplementary figures and images for: Repeated exposure to nanosecond high power pulsed microwaves increases cancer incidence in rat
Source: PLoS One. 2020 Apr 8;15(4):e0226858. doi: 10.1371/journal.pone.0226858 (PMC7141660; doi:10.1371/journal.pone.0226858)

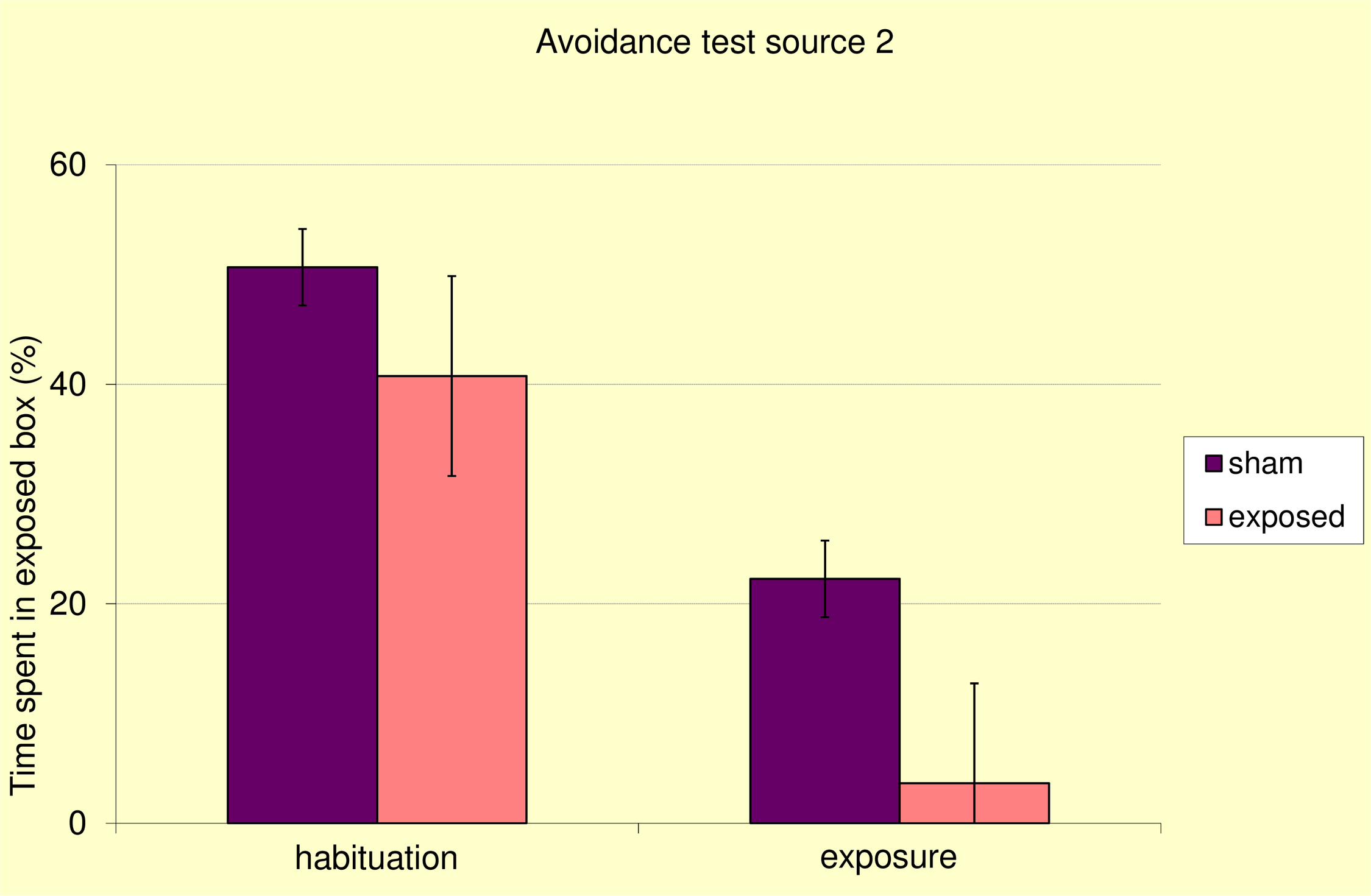

Supplement: S1 Fig — (TIF) [file pone.0226858.s001.tif]
