# Supplementary material for: Repeated exposure to nanosecond high power pulsed microwaves increases cancer incidence in rat
Source: PLoS One. 2020 Apr 8;15(4):e0226858. doi: 10.1371/journal.pone.0226858 (PMC7141660; doi:10.1371/journal.pone.0226858)
Supplement: S1 Table — (PDF) [file pone.0226858.s002.pdf]

19 et 20 dec 06	Animal #	Training			Test		
		**Trial 1**	**Trial 2**	**Trial 3**	**Trial 1**	**Trial 2**	**Trial 3**
**Sham**	25	2	17	7	6	**60**	**60**
	26	4	31	12	7	8	10
	27	8	2	10	59	**60**	**60**
	28	18	20	2	46	**60**	**60**
	29	5	14	4	13	4	44
	30	10	**60**	**60**	10	**60**	**60**
	31	11	7	8	10	**60**	**60**
	32	3	8	39	13	26	13
	33	4	5	6	32	**60**	**60**
	34	8	3	10	1	15	4
	35	33	12	30	25	17	7
	36	2	4	10	2	9	**60**
	37	9	3	11	45	17	5
	38	36	11	10	8	**60**	**60**
	39	33	2	37	40	31	26
	40	48	3	**60**	10	**60**	**60**
	41	12	8	30	27	26	**60**
	42	19	10	8	7	3	4
	43	3	27	15	19	20	17
	44	1	41	31	47	**60**	**60**
	45	**60**	**60**	**60**	**60**	**60**	**60**
	46	14	9	11	9	6	16
	47	20	10	4	7	doesn't stay on the rod	
	48	1	**60**	37	14	12	24

	Cible	Training			Test		
		**Trial 1**	**Trial 2**	**Trial 3**	**Trial 1**	**Trial 2**	**Trial 3**
**Exposés**	1	11	**60**	**60**	7	**60**	**60**
	2	22	13	**60**	**60**	46	**60**
	3	1	22	20	14	**60**	**60**
	4	1	10	1	11	27	7
	5	1	**60**	24	**60**	**60**	**60**
	6	22	20	**60**	**60**	**60**	**60**
	7	2	**60**	**60**	**60**	14	**60**
	8	2	**60**	**60**	**60**	**60**	**60**
	9	3	**60**	**60**	**60**	**60**	**60**
	10	2	24	17	**60**	**60**	**60**
	11	21	**60**	**60**	27	**60**	**60**
	12	31	**60**	**60**	34	**60**	**60**
	13	8	28	45	45	**60**	**60**
	14	6	**60**	26	30	**60**	**60**
	15	1	57	44	**60**	**60**	**60**
	16	2	33	22	48	**60**	**60**
	17	1	30	33	don't stay on the rod		
	18	1	10	27			
	19	8	42	**60**	14	**60**	**60**
	20	6	**60**	**60**	55	**60**	37
	21	20	16	15	8	34	2
	22	8	11	6	6	15	7
	23	1	33	15	9	37	35
	24	1	**60**	**60**	21	**60**	**60**

	Training			Test	
	failure	success		failure	success
Ex	65	7	Ex	49	23
Te	49	23	Te	31	41
	p	2,0E-10		p	0,000005
