# Supplementary material for: Repeated exposure to nanosecond high power pulsed microwaves increases cancer incidence in rat
Source: PLoS One. 2020 Apr 8;15(4):e0226858. doi: 10.1371/journal.pone.0226858 (PMC7141660; doi:10.1371/journal.pone.0226858)
Supplement: S2 Table — (PDF) [file pone.0226858.s003.pdf]

Avoidance test source 2

| | | Time spent in non-shielded side over 300s | Time spent in non-shielded side over 900s | % time spent in non-shielded side | | Start hab | Start exp |
|---|---|---|---|---|---|---|---|
| | | 5 min habituation | 15 min exposure | 5 min habituation | 15 min exposure | | |
| Sham | 1 | 180 | 215 | 60 | 23,9 | NS | NS |
| | 2 | 180 | 330 | 60 | 36,6 | NS | NS |
| | 3 | 180 | 180 | 60 | 20 | S | S |
| | 4 | 111 | 280 | 37 | 31,1 | S | S |
| | 5 | 180 | 153 | 60 | 17 | NS | S |
| | 6 | 168 | 40 | 56 | 4,4 | S | NS |
| | 7 | 129 | 315 | 43 | 35 | NS | S |
| | 8 | 129 | 130 | 43 | 14,4 | S | S |
| | 9 | 150 | 156 | 50 | 17,3 | NS | S |
| | 10 | 159 | 190 | 53 | 21,1 | S | NS |
| | 11 | 129 | 220 | 43 | 24,4 | NS | NS |
| | 12 | 129 | 160 | 43 | 17,7 | S | S |
| Av | | **152,0** | **197,4** | **50,7** | **21,9** | | |
| SD | | **25,7** | **81,9** | **8,6** | **9,1** | | |
| Exposed | 1 | 30 | 5 | 10 | 0,56 | NS | S |
| | 2 | 129 | 52 | 43 | 5,7 | NS | S |
| | 3 | 168 | 95 | 56 | 10,5 | NS | NS |
| | 4 | 129 | 62 | 43 | 6,8 | NS | S |
| | 5 | 39 | 7 | 13 | 0,8 | NS | S |
| | 6 | 138 | 18 | 46 | 2 | NS | S |
| | 7 | 123 | 18 | 41 | 2 | NS | NS |
| | 8 | 138 | 21 | 46 | 2,3 | NS | NS |
| | 9 | 138 | 14 | 46 | 1,6 | NS | NS |
| | 10 | 165 | 15 | 55 | 1,7 | NS | S |
| | 11 | 141 | 84 | 47 | 9,3 | NS | NS |
| | 12 | 129 | 8 | 43 | 0,9 | NS | NS |
| Av | | **122,3** | **33,3** | **40,8** | **3,7** | | |
| SD | | **43,3** | **31,6** | **14,4** | **3,5** | | |

**NS: start in non-shielded side**
**S: start in shielded side**
