# Supplementary material for: Repeated exposure to nanosecond high power pulsed microwaves increases cancer incidence in rat
Source: PLoS One. 2020 Apr 8;15(4):e0226858. doi: 10.1371/journal.pone.0226858 (PMC7141660; doi:10.1371/journal.pone.0226858)
Supplement: S3 Table — Magnification x10. (PDF) [file pone.0226858.s004.pdf]

Nom de l'image	Calibration	DO min	DO max	Surface	Surface marquée	Ratio	Nombre		rat	groupe	région
FC_01-03-1	Objectif_10	0	104	143822,1	11291,15	0,08	28727	rat1 gp3 s1	1	3	FC
DG_01-03-1	Objectif_10	0	132	21861,05	424,1	0,02	1079	rat1 gp3 s1	1	3	DG
CCx_01-03-1	Objectif_10	0	64	35339,13	8768,56	0,25	22309	rat1 gp3 s1	1	3	CCx
CF-02-03-1	Objectif_10	0	111	69911,42	6613,85	0,09	16827	rat2 gp3 s1	2	3	CF
Cpu_02-03-1	Objectif_10	0	111	144419,6	8276,06	0,06	21056	rat2 gp3 s1	2	3	Cpu
LGP_02-03-1	Objectif_10	0	90	76930,12	10559,68	0,14	26866	rat2 gp3 s1	2	3	LGP
CF_04-03-1	Objectif_10	0	115	136203,6	19101,05	0,14	48597	rat4 gr3 s1	4	3	CF
DG_04-03-1	Objectif_10	0	84	20607,62	2948,27	0,14	7501	rat4 gr3 s1	4	3	DG
CPu_04-03-1	Objectif_10	0	106	151791,2	15819,87	0,1	40249	rat4 gr3 s1	4	3	Cpu
LGP_04-03-1	Objectif_10	0	94	111162,8	21301,35	0,19	54195	rat4 gr3 s1	4	3	LGP
CCx_04-03-1	Objectif_10	0	106	34714,57	6504,98	0,19	16550	rat4 gr3 s1	4	3	CCx
CF_05-03-1	Objectif_10	0	89	158792,6	29243,71	0,18	74402	rat5 gr3 s1	5	3	CF
DG_05-03-1	Objectif_10	0	92	19677,66	7338,64	0,37	18671	rat5 gr3 s1	5	3	DG
CPu_05-03-1	Objectif_10	0	130	161466,9	53679,24	0,33	136571	rat5 gr3 s1	5	3	Cpu
LGP_05-03-1	Objectif_10	0	95	70003,01	9045,65	0,13	23014	rat5 gr3 s1	5	3	LGP
CCx_05-03-1	Objectif_10	0	181	114467,6	49461,03	0,43	125839	rat5 gr3 s1	5	3	CCx
CF_06-03-1	Objectif_10	0	140	130719	28841,23	0,22	73378	rat6 gr3 s1	6	3	CF
DG_06-03-1	Objectif_10	0	167	17971,43	5218,92	0,29	13278	rat6 gr3 s1	6	3	DG
CPu_06-03-1	Objectif_10	0	149	135661,2	49029,07	0,36	124740	rat6 gr3 s1	6	3	Cpu
LGP_06-03-1	Objectif_10	0	170	100394,8	15978,27	0,16	40652	rat6 gr3 s1	6	3	LGP
CCx_06-03-1	Objectif_10	0	104	90978,91	56704,16	0,62	144267	rat6 gr3 s1	6	3	CCx
CF_07-03-1	Objectif_10	0	108	142658,7	29022,82	0,2	73840	rat7 gr3 s1	7	3	CF
DG_07-03-1	Objectif_10	0	81	20822,61	5388,32	0,26	13709	rat7 gr3 s1	7	3	DG
CPu_07-03-1	Objectif_10	0	90	160032,7	35704,28	0,22	90839	rat7 gr3 s1	7	3	Cpu
LGP_07-03-1	Objectif_10	0	93	66392,05	18930,47	0,29	48163	rat7 gr3 s1	7	3	LGP
CCx_07-03-1	Objectif_10	0	193	77577,48	32936,42	0,42	83797	rat7 gr3 s1	7	3	CCx
CF_08-03-1	Objectif_10	0	142	139249,4	28593,21	0,21	72747	rat8 gr3 s1	8	3	CF
DG_08-03-1	Objectif_10	0	82	21425,95	6513,23	0,3	16571	rat8 gr3 s1	8	3	DG
CPu_08-03-1	Objectif_10	0	148	160084,2	31182,23	0,19	79334	rat8 gr3 s1	8	3	Cpu
LGP_08-03-1	Objectif_10	0	110	115242,3	26495,51	0,23	67410	rat8 gr3 s1	8	3	LGP
CCx_08-03-1	Objectif_10	0	147	77604,98	47361,75	0,61	120498	rat8 gr3 s1	8	3	CCx
CF_09-03-1	Objectif_10	0	104	154885,7	24075,1	0,16	61252	rat9 gr3 s1	9	3	CF
DG_09-03-1	Objectif_10	0	83	14558,58	4762,59	0,33	12117	rat9 gr3 s1	9	3	DG
CPu_09-03-1	Objectif_10	0	124	160238,7	36475,44	0,23	92801	rat9 gr3 s1	9	3	Cpu
LGP_09-03-1	Objectif_10	0	84	102809,3	26211,72	0,25	66688	rat9 gr3 s1	9	3	LGP
CCx_09-03-1	Objectif_10	0	110	108155,6	23305,9	0,22	59295	rat9 gr3 s1	9	3	CCx
CF_10-03-1	Objectif_10	0	166	164844	40984,51	0,25	104273	rat10 gr3 s1	10	3	CF
DG_10-03-1	Objectif_10	0	96	11262,46	3458,84	0,31	8800	rat10 gr3 s1	10	3	DG
CPu_10-03-1	Objectif_10	0	147	162076,6	52122,77	0,32	132611	rat10 gr3 s1	10	3	Cpu
LGP_10-03-1	Objectif_10	0	82	77205,65	22677,02	0,29	57695	rat10 gr3 s1	10	3	LGP
CCx_10-03-1	Objectif_10	0	110	102830,2	47842,05	0,47	121720	rat10 gr3 s1	10	3	CCx
CF_11-03-1	Objectif_10	0	112	168468	46289,51	0,27	117770	rat11 gr3 s1	11	3	CF
DG_11-03-1	Objectif_10	0	115	22076,05	9301,92	0,42	23666	rat11 gr3 s1	11	3	DG
CPu_11-03-1	Objectif_10	0	160	165759,4	38530,7	0,23	98030	rat11 gr3 s1	11	3	Cpu
LGP_11-03-1	Objectif_10	0	97	53058,62	16953,04	0,32	43132	rat11 gr3 s1	11	3	LGP
CCx_11-03-1	Objectif_10	0	93	93790,79	52156,57	0,56	132697	rat11 gr3 s1	11	3	CCx
CF_12-03-1	Objectif_10	0	74	164770,5	62195,07	0,38	158237	rat12 gr3 s1	12	3	CF
DG_12-03-1	Objectif_10	0	40	24379,72	10161,13	0,42	25852	rat12 gr3 s1	12	3	DG
CPu_12-03-1	Objectif_10	0	102	168833,5	32750,5	0,19	83324	rat12 gr3 s1	12	3	Cpu
LGP_12-03-1	Objectif_10	0	95	71234,82	26311,17	0,37	66941	rat12 gr3 s1	12	3	LGP
CCx_12-03-1	Objectif_10	0	49	107290,1	40773,05	0,38	103735	rat12 gr3 s1	12	3	CCx
CF_13-03-1	Objectif_10	0	132	113291,6	31974,62	0,28	81350	rat13 gr3 s1	13	3	CF
DG_13-03-1	Objectif_10	0	106	15212,61	6510,88	0,43	16565	rat13 gr3 s1	13	3	DG
CPu_13-03-1	Objectif_10	0	157	152248,7	35291,18	0,23	89788	rat13 gr3 s1	13	3	Cpu
LGP_13-03-1	Objectif_10	0	176	64141,84	15149,72	0,24	38544	rat13 gr3 s1	13	3	LGP
CCx_13-03-1	Objectif_10	0	76	107797,5	46061,14	0,43	117189	rat13 gr3 s1	13	3	CCx
CF_14-03-S1	Objectif_10	0	110	162399,6	43189,91	0,27	109884	rat 14 gr3 S1	14	3	CF
DG_14-03-S1	Objectif_10	0	108	13746,93	5292,81	0,39	13466	rat 14 gr3 S1	14	3	DG
CPu_14-03-S1	Objectif_10	0	155	126941,8	25718,45	0,2	65433	rat 14 gr3 S1	14	3	Cpu
LGP_14-03-S1	Objectif_10	0	161	102128,2	25188,22	0,25	64084	rat 14 gr3 S1	14	3	LGP
CCx_14-03-S1	Objectif_10	0	132	79090,72	24517,68	0,31	62378	rat 14 gr3 S1	14	3	CCx
CF_15-03-S1	Objectif_10	0	143	156930	50303,34	0,32	127982	rat 15 gr3 S1	15	3	CF
DG_15-03-S1	Objectif_10	0	79	27626,7	12138,17	0,44	30882	rat 15 gr3 S1	15	3	DG
CPu_15-03-S1	Objectif_10	0	156	117215	21366,99	0,18	54362	rat 15 gr3 S1	15	3	Cpu
LGP_15-03-S1	Objectif_10	0	140	100364,6	29765,29	0,3	75729	rat 15 gr3 S1	15	3	LGP
CCx_15-03-S1	Objectif_10	0	86	117664,3	63123,05	0,54	160598	rat 15 gr3 S1	15	3	CCx

CF_16-03-1	Objectif_10	0	130	129311,1	48082,6	0,37	122332	rat 16 gr3 S1	16	3	CF
DG_16-03-1	Objectif_10	0	81	16915,7	8665,58	0,51	22047	rat 16 gr3 S1	16	3	DG
CPu_16-03-1	Objectif_10	0	146	145500,1	50345	0,35	128088	rat 16 gr3 S1	16	3	Cpu
LGP_16-03-1	Objectif_10	0	123	59320,3	18452,92	0,31	46948	rat 16 gr3 S1	16	3	LGP
CCx_16-03-1	Objectif_10	0	80	54209,47	31876,36	0,59	81100	rat 16 gr3 S1	16	3	CCx
CF_17-03-1	Objectif_10	0	136	160448,5	38676,52	0,24	98401	rat 17 gr3 S1	17	3	CF
DG_17-03-1	Objectif_10	0	102	30090,73	13948,17	0,46	35487	rat 17 gr3 S1	17	3	DG
CPu_17-03-1	Objectif_10	0	147	116479,2	41354,37	0,36	105214	rat 17 gr3 S1	17	3	Cpu
LGP_17-03-1	Objectif_10	0	116	80597,27	28236,32	0,35	71839	rat 17 gr3 S1	17	3	LGP
CCx_17-03-1	Objectif_10	0	85	167723,5	68564,45	0,41	174442	rat 17 gr3 S1	17	3	CCx
CF_18-03-1	Objectif_10	0	171	169234,8	54437,04	0,32	138499	rat 18 gr3 S1	18	3	CF
DG_18-03-1	Objectif_10	0	106	30982,96	13239,11	0,43	33683	rat 18 gr3 S1	18	3	DG
CPu_18-03-1	Objectif_10	0	171	114653,5	34279,86	0,3	87215	rat 18 gr3 S1	18	3	Cpu
LGP_18-03-1	Objectif_10	0	144	71588,18	19023,23	0,27	48399	rat 18 gr3 S1	18	3	LGP
CCx_18-03-1	Objectif_10	0	115	98496,38	36993,88	0,38	94120	rat 18 gr3 S1	18	3	CCx
CF_19-03-1	Objectif_10	0	49	121708,7	42915,57	0,35	109186	rat19 gr3 S1	19	3	CF
DG_19-03-1	Objectif_10	0	27	36070,99	15341,53	0,43	39032	rat19 gr3 S1	19	3	DG
CPu_19-03-1	Objectif_10	0	89	97964,98	46080,8	0,47	117239	rat19 gr3 S1	19	3	Cpu
LGP_19-03-1	Objectif_10	0	29	61913,64	26608,31	0,43	67697	rat19 gr3 S1	19	3	LGP
CF_20-03-1	Objectif_10	0	131	136559,4	35504,61	0,26	90331	rat20 gr3 S1	20	3	CF
DG_20-03-1	Objectif_10	0	98	26118,96	10196,11	0,39	25941	rat20 gr3 S1	20	3	DG
CPu_20-03-1	Objectif_10	0	149	130210	46405,85	0,36	118066	rat20 gr3 S1	20	3	Cpu
LGP_20-03-1	Objectif_10	0	126	92679,63	35272,71	0,38	89741	rat20 gr3 S1	20	3	LGP
CCx_20-03-1	Objectif_10	0	120	169746,5	74483,77	0,44	189502	rat20 gr3 S1	20	3	CCx
CF_21-03-1	Objectif_10	0	128	151562,5	50152,01	0,33	127597	rat21 gr3 S1	21	3	CF
DG_21-03-1	Objectif_10	0	90	29005,92	13633,73	0,47	34687	rat21 gr3 S1	21	3	DG
Cpu_21-03-1	Objectif_10	0	131	110579,1	43415,92	0,39	110459	rat21 gr3 S1	21	3	Cpu
LGP_21-03-1	Objectif_10	0	91	72318,86	28465,08	0,39	72421	rat21 gr3 S1	21	3	LGP
CCx_21-03-1	Objectif_10	0	76	125971,4	53805,02	0,43	136891	rat21 gr3 S1	21	3	CCx
CF_22-03-1	Objectif_10	0	124	169206,9	48236,29	0,29	122723	rat22 gr3 S1	22	3	CF
DG_22-03-1	Objectif_10	0	106	20711,77	7904,63	0,38	20111	rat22 gr3 S1	22	3	DG
Cpu_22-03-1	Objectif_10	0	144	133775	39669,76	0,3	100928	rat22 gr3 S1	22	3	Cpu
LGP_22-03-1	Objectif_10	0	94	76680,53	23959,94	0,31	60959	rat22 gr3 S1	22	3	LGP
CCx_22-03-1	Objectif_10	0	82	111951,7	63461,08	0,57	161458	rat22 gr3 S1	22	3	CCx
CF_24-03-1	Objectif_10	0	112	169955,3	51712,81	0,3	131568	rat24 gr3 S1	24	3	CF
DG_24-03-1	Objectif_10	0	81	22276,51	11346,18	0,51	28867	rat24 gr3 S1	24	3	DG
CPu_24-03-1	Objectif_10	0	135	105671,9	30036,49	0,28	76419	rat24 gr3 S1	24	3	Cpu
LGP_24-03-1	Objectif_10	0	124	112976	37154,24	0,33	94528	rat24 gr3 S1	24	3	LGP
CCx_24-03-1	Objectif_10	0	54	141257,1	52145,17	0,37	132668	rat24 gr3 S1	24	3	CCx
CF_25-03-1	Objectif_10	0	157	169526	68117,94	0,4	173306	rat25 gr3 S1	25	3	CF
DG_25-03-1	Objectif_10	0	95	39174,52	16593	0,42	42216	rat25 gr3 S1	25	3	DG
CPu_25-03-1	Objectif_10	0	149	157298,3	64110,79	0,41	163111	rat25 gr3 S1	25	3	Cpu
LGP_25-03-1	Objectif_10	0	150	94832,77	43520,86	0,46	110726	rat25 gr3 S1	25	3	LGP
CCx_25-03-1	Objectif_10	0	89	158550,1	68687,47	0,43	174755	rat25 gr3 S1	25	3	CCx
CCx_01-04-1	Objectif_10	0	135	126270,9	40249,51	0,32	102403	rat1 gr4 S1	1	4	CCx
CCx_02-04-1	Objectif_10	0	149	92960,66	34874,16	0,38	88727	rat2 gr4 S1	2	4	CCx
CCx_03-04-1	Objectif_10	0	110	107640,3	32836,19	0,31	83542	rat3 gr4 S1	3	4	CCx
CCx_04-04-1	Objectif_10	0	119	114449,5	41671,96	0,36	106022	rat4 gr4 S1	4	4	CCx
CCx_05-04-1	Objectif_10	0	104	134791,8	51285,96	0,38	130482	rat5 gr4 S1	5	4	CCx
CCx_06-04-1	Objectif_10	0	100	90117,73	25083,28	0,28	63817	rat6 gr4 S1	6	4	CCx
CF_02-04-1	Objectif_10	0	201	163447,5	44165,07	0,27	112365	rat2 gr4 S1	2	4	CF
CF_03-04-1	Objectif_10	0	155	138269,9	15105,7	0,11	38432	rat3 gr4 S1	3	4	CF
CF_04-04-1	Objectif_10	0	146	168455,4	23208,04	0,14	59046	rat4 gr4 S1	4	4	CF
CF_05-04-1	Objectif_10	0	158	152015,7	19598,66	0,13	49863	rat5 gr4 S1	5	4	CF
CF_06-04-1	Objectif_10	0	131	165444,6	12145,64	0,07	30901	rat6 gr4 S1	6	4	CF
CPu_01-04-1	Objectif_10	0	200	125017	15574,61	0,12	39625	rat1 gr4 S1	1	4	Cpu
CPu_02-04-1	Objectif_10	0	200	152635,1	2800,09	0,02	7124	rat2 gr4 S1	2	4	Cpu
CPu_03-04-1	Objectif_10	0	143	153985,6	17366,13	0,11	44183	rat3 gr4 S1	3	4	Cpu
CPu_04-04-1	Objectif_10	0	157	137352,1	9959,89	0,07	25340	rat4 gr4 S1	4	4	Cpu
Cpu_05-04-1	Objectif_10	0	152	147173,3	12586,25	0,09	32022	rat5 gr4 S1	5	4	Cpu
CPu_06-04-1	Objectif_10	0	149	165444,6	12500,17	0,08	31803	rat6 gr4 S1	6	4	Cpu
DG_01-04-1	Objectif_10	0	129	28481,2	9318,43	0,33	23708	rat1 gr4 S1	1	4	DG
DG_02-04-1	Objectif_10	0	125	19349,07	9935,91	0,51	25279	rat2 gr4 S1	2	4	DG
DG_03-04-1	Objectif_10	0	100	18930,08	8419,53	0,44	21421	rat3 gr4 S1	3	4	DG
DG_04-04-1	Objectif_10	0	126	25858,37	5112,4	0,2	13007	rat4 gr4 S1	4	4	DG
DG_05-04-1	Objectif_10	0	136	27303,62	7652,29	0,28	19469	rat5 gr4 S1	5	4	DG
DG_06-04-1	Objectif_10	0	116	26717,19	5621,01	0,21	14301	rat6 gr4 S1	6	4	DG
FC_01-04-1	Objectif_10	0	188	166803	28285,85	0,17	71965	rat1 gr4 S1	1	4	FC

LGP_01-04-1	Objectif_10	0	176	84542,71	18702,11	0,22	47582	rat1 gr4 S1	1	4	LGP
LGP_02-04-1	Objectif_10	0	207	107395,4	9432,81	0,09	23999	rat2 gr4 S1	2	4	LGP
LGP_03-04-1	Objectif_10	0	111	64310,46	10596,24	0,16	26959	rat3 gr4 S1	3	4	LGP
LGP_04-04-1	Objectif_10	0	141	52837,33	9646,63	0,18	24543	rat4 gr4 S1	4	4	LGP
LGP_05-04-1	Objectif_10	0	131	74975,88	18029,6	0,24	45871	rat5 gr4 S1	5	4	LGP
LGP_06-04-1	Objectif_10	0	138	91075,2	18120,39	0,2	46102	rat6 gr4 S1	6	4	LGP
CF_07-04-1	Objectif_10	0	133	165100,3	24063,71	0,15	61223	rat7 gr4 S1	7	4	CF
DG_07-04-1	Objectif_10	0	97	26388,99	8101,55	0,31	20612	rat7 gr4 S1	7	4	DG
CPu_07-04-1	Objectif_10	0	114	159230,5	13010,35	0,08	33101	rat7 gr4 S1	7	4	Cpu
LGP_07-04-1	Objectif_10	0	100	80141,34	15523,91	0,19	39496	rat7 gr4 S1	7	4	LGP
CCx_07-04-1	Objectif_10	0	99	164763,1	50169,3	0,3	127641	rat7 gr4 S1	7	4	CCx
CF_08-04-1	Objectif_10	0	169	153083,2	16721,92	0,11	42544	rat8 gr4S1	8	4	CF
DG_08-04-1	Objectif_10	0	92	26379,16	6268,36	0,24	15948	rat8 gr4S1	8	4	DG
CPu_08-04-1	Objectif_10	0	97	100919,9	8053,99	0,08	20491	rat8 gr4S1	8	4	Cpu
CCx_08-04-1	Objectif_10	0	75	136158	23046,88	0,17	58636	rat8 gr4S1	8	4	CCx
CF_09-04-1	Objectif_10	0	123	138501,8	5235,03	0,04	13319	rat9 gr4 S1	9	4	CF
DG_09-04-1	Objectif_10	0	97	25143,41	6900,39	0,27	17556	rat9 gr4 S1	9	4	DG
CPu_09-04-1	Objectif_10	0	122	136168,3	5416,23	0,04	13780	rat9 gr4 S1	9	4	Cpu
LGP_09-04-1	Objectif_10	0	112	69726,3	11162,62	0,16	28400	rat9 gr4 S1	9	4	LGP
CCx_09-04-1	Objectif_10	0	72	53097,14	9407,26	0,18	23934	rat9 gr4 S1	9	4	CCx
CF_10-04-1	Objectif_10	0	115	134752,1	8079,15	0,06	20555	rat10 gr4 S1	10	4	CF
DG_10-04-1	Objectif_10	0	97	20220,07	3390,84	0,17	8627	rat10 gr4 S1	10	4	DG
CPu_10-04-1	Objectif_10	0	128	147014,1	22721,83	0,15	57809	rat10 gr4 S1	10	4	Cpu
LGP_10-04-1	Objectif_10	0	128	64240,5	13624,29	0,21	34663	rat10 gr4 S1	10	4	LGP
CCx_10-04-1	Objectif_10	0	132	28792,49	8632,56	0,3	21963	rat10 gr4 S1	10	4	CCx
CF_11-04-1	Objectif_10	0	140	152273,5	15947,22	0,1	40573	rat11 gr4 S1	11	4	CF
DG_11-04-1	Objectif_10	0	92	27436,07	7341,39	0,27	18678	rat11 gr4 S1	11	4	DG
CPu_11-04-1	Objectif_10	0	134	111236,7	8443,5	0,08	21482	rat11 gr4 S1	11	4	Cpu
LGP_11-04-1	Objectif_10	0	105	73599,8	12430,6	0,17	31626	rat11 gr4 S1	11	4	LGP
CCx_11-04-1	Objectif_10	0	92	79992,77	30870,94	0,39	78542	rat11 gr4 S1	11	4	CCx
CF_12-04-1	Objectif_10	0	182	144306,8	17431,38	0,12	44349	rat12 gr4 S1	12	4	CF
DG_12-04-1	Objectif_10	0	103	30584,41	8325,98	0,27	21183	rat12 gr4 S1	12	4	DG
CPu_12-04-1	Objectif_10	0	139	134377,5	7235,66	0,05	18409	rat12 gr4 S1	12	4	Cpu
LGP_12-04-1	Objectif_10	0	116	57943,84	8331,09	0,14	21196	rat12 gr4 S1	12	4	LGP
CCx_12-04-1	Objectif_10	0	114	112768,4	45520,31	0,4	115813	rat12 gr4 S1	12	4	CCx
CF_13-04-1	Objectif_10	0	161	125081,1	20464,54	0,16	52066	rat13 gr4 S1	13	4	CF
DG_13-04-1	Objectif_10	0	108	18340,9	4475,66	0,24	11387	rat13 gr4 S1	13	4	DG
CPu_13-04-1	Objectif_10	0	179	120794,9	9639,55	0,08	24525	rat13 gr4 S1	13	4	Cpu
LGP_13-04-1	Objectif_10	0	177	85354,76	12890,86	0,15	32797	rat13 gr4 S1	13	4	LGP
CCx_13-04-1	Objectif_10	0	128	120065	31994,67	0,27	81401	rat13 gr4 S1	13	4	CCx
CF_14-04-1	Objectif_10	0	160	149134,2	18558,65	0,12	47217	rat14 gr4 S1	14	4	CF
DG_14-04-1	Objectif_10	0	169	25463,75	8830,66	0,35	22467	rat14 gr4 S1	14	4	DG
CPu_14-04-1	Objectif_10	0	165	102254,3	6217,27	0,06	15818	rat14 gr4 S1	14	4	Cpu
LGP_14-04-1	Objectif_10	0	159	66380,27	9785,77	0,15	24897	rat14 gr4 S1	14	4	LGP
CCx_14-04-1	Objectif_10	0	134	104153,9	31706,96	0,3	80669	rat14 gr4 S1	14	4	CCx
CF_15-04-1	Objectif_10	0	154	106896,6	20287,67	0,19	51616	rat15 gr4 S1	15	4	CF
DG_15-04-1	Objectif_10	0	123	21451,1	4967,37	0,23	12638	rat15 gr4 S1	15	4	DG
CPu_15-04-1	Objectif_10	0	149	141169	9589,24	0,07	24397	rat15 gr4 S1	15	4	Cpu
LGP_15-04-1	Objectif_10	0	137	62398,66	13841,65	0,22	35216	rat15 gr4 S1	15	4	LGP
CCx_15-04-1	Objectif_10	0	104	143725	64032,97	0,45	162913	rat15 gr4 S1	15	4	CCx
CF_17-04-1	Objectif_10	0	159	160423	17557,94	0,11	44671	rat17 gr4 S1	17	4	CF
DG_17-04-1	Objectif_10	0	106	29964,57	6184,64	0,21	15735	rat17 gr4 S1	17	4	DG
CPu_17-04-1	Objectif_10	0	170	130172,7	21795,41	0,17	55452	rat17 gr4 S1	17	4	Cpu
LGP_17-04-1	Objectif_10	0	149	93078,59	14682,78	0,16	37356	rat17 gr4 S1	17	4	LGP
CCx_17-04-1	Objectif_10	0	128	121309	34754,67	0,29	88423	rat17 gr4 S1	17	4	CCx
CF_19-04-1	Objectif_10	0	157	147011,3	25194,51	0,17	64100	rat19 gr4 S1	19	4	CF
DG_19-04-1	Objectif_10	0	138	28693,05	7634,21	0,27	19423	rat19 gr4 S1	19	4	DG
Cpu_19-04-1	Objectif_10	0	168	149455,3	15273,93	0,1	38860	rat19 gr4 S1	19	4	Cpu
LGP_19-04-1	Objectif_10	0	148	78453,98	13819,64	0,18	35160	rat19 gr4 S1	19	4	LGP
CCx_19-04-1	Objectif_10	0	146	82876,18	26043,89	0,31	66261	rat19 gr4 S1	19	4	CCx
CF_20-04-1	Objectif_10	0	144	110829,5	23592,83	0,21	60025	rat20 gr4 S1	20	4	CF
DG_20-04-1	Objectif_10	0	98	27574,43	6019,56	0,22	15315	rat20 gr4 S1	20	4	DG
CPu_20-04-1	Objectif_10	0	151	100572,1	5542,4	0,06	14101	rat20 gr4 S1	20	4	Cpu
LGP_20-04-1	Objectif_10	0	147	82366,79	13699,76	0,17	34855	rat20 gr4 S1	20	4	LGP
CCx_20-04-1	Objectif_10	0	94	74091,52	19347,89	0,26	49225	rat20 gr4 S1	20	4	CCx
CF_21-04-1	Objectif_10	0	144	131948,5	15590,72	0,12	39666	rat21 gr4 S1	21	4	CF
DG_21-04-1	Objectif_10	0	101	16955,39	4405,7	0,26	11209	rat21 gr4 S1	21	4	DG
CPu_21-04-1	Objectif_10	0	145	125154,2	15070,72	0,12	38343	rat21 gr4 S1	21	4	Cpu

LGP_21-04-1	Objectif_10	0	144	94368,18	13025,29	0,14	33139	rat21 gr4 S1	21	4	LGP
CCx_21-04-1	Objectif_10	0	127	138767,5	39392,66	0,28	100223	rat21 gr4 S1	21	4	CCx
CF_22-04-1	Objectif_10	0	143	122469,3	13061,45	0,11	33231	rat22 gr4 S1	22	4	CF
DG_22-04-1	Objectif_10	0	106	22532,38	6605,21	0,29	16805	rat22 gr4 S1	22	4	DG
CPu_22-04-1	Objectif_10	0	148	114643,3	14329,43	0,12	36457	rat22 gr4 S1	22	4	Cpu
LGP_22-04-1	Objectif_10	0	98	76461,21	10211,44	0,13	25980	rat22 gr4 S1	22	4	LGP
CCx_22-04-1	Objectif_10	0	89	116565,3	33310,21	0,29	84748	rat22 gr4 S1	22	4	CCx
CF_23-04-1	Objectif_10	0	167	130537,4	12627,91	0,1	32128	rat23 gr4 S1	23	4	CF
DG_23-04-1	Objectif_10	0	108	27399,52	6400,43	0,23	16284	rat23 gr4 S1	23	4	DG
CPu_23-04-1	Objectif_10	0	158	114026,6	4745,69	0,04	12074	rat23 gr4 S1	23	4	Cpu
LGP_23-04-1	Objectif_10	0	138	56438,85	7412,92	0,13	18860	rat23 gr4 S1	23	4	LGP
CCx_23-04-1	Objectif_10	0	98	111619,5	34230,34	0,31	87089	rat23 gr4 S1	23	4	CCx
CF_24-04-1	Objectif_10	0	150	80905,43	10490,11	0,13	26689	rat24 gr4 S1	24	4	CF
DG_24-04-1	Objectif_10	0	98	23531,12	4388,4	0,19	11165	rat24 gr4 S1	24	4	DG
CPu_24-04-1	Objectif_10	0	154	135888,8	7370,87	0,05	18753	rat24 gr4 S1	24	4	Cpu
LGP_24-04-1	Objectif_10	0	143	78640,28	11072,22	0,14	28170	rat24 gr4 S1	24	4	LGP
CCx_24-04-1	Objectif_10	0	113	107275,9	36813,86	0,34	93662	rat24 gr4 S1	24	4	CCx
CF_01-05-1	Objectif_10	0	173	84305,31	18963,49	0,22	48247	rat1 gr5 S1	1	5	CF
DG_01-05-1	Objectif_10	0	101	18629	4997,24	0,27	12714	rat1 gr5 S1	1	5	DG
CPu_01-05-1	Objectif_10	0	166	51489,95	13369,99	0,26	34016	rat1 gr5 S1	1	5	Cpu
CCx_01-05-1	Objectif_10	0	143	50536,81	17540,65	0,35	44627	rat1 gr5 S1	1	5	CCx
CF_02-05-1	Objectif_10	0	132	77320,42	16886,22	0,22	42962	rat2 gr5 S1	2	5	CF
DG_02-05-1	Objectif_10	0	89	16118,2	4834,52	0,3	12300	rat2 gr5 S1	2	5	DG
Cpu_02-05-1	Objectif_10	0	126	89612,27	12881,04	0,14	32772	rat2 gr5 S1	2	5	Cpu
LGP_02-05-1	Objectif_10	0	123	50496,32	8847,95	0,18	22511	rat2 gr5 S1	2	5	LGP
CCx_02-05-1	Objectif_10	0	72	28621,91	10250,75	0,36	26080	rat2 gr5 S1	2	5	CCx
CF_03-05-1	Objectif_10	0	107	41116,18	9467	0,23	24086	rat3 gr5 S1	3	5	CF
Cpu_03-05-1	Objectif_10	0	136	49372,2	6225,13	0,13	15838	rat3 gr5 S1	3	5	Cpu
LGP_03-05-1	Objectif_10	0	136	54279,82	12136,21	0,22	30877	rat3 gr5 S1	3	5	LGP
CCx_03-05-1	Objectif_10	0	95	44412,7	19031,48	0,43	48420	rat3 gr5 S1	3	5	CCx
CF_04-05-1	Objectif_10	0	200	27643,21	1128,84	0,04	2872	rat4 gr5 S1	4	5	CF
DG_04-05-1	Objectif_10	0	157	23468,63	5305,39	0,23	13498	rat4 gr5 S1	4	5	DG
CPu_04-05-1	Objectif_10	0	174	42529,2	2050,94	0,05	5218	rat4 gr5 S1	4	5	Cpu
LGP_04-05-1	Objectif_10	0	106	47102,34	8577,53	0,18	21823	rat4 gr5 S1	4	5	LGP
CCx_04-05-1	Objectif_10	0	132	30463,74	989,7	0,03	2518	rat4 gr5 S1	4	5	CCx
CF_05-05-1	Objectif_10	0	117	71301,25	7800,08	0,11	19845	rat5 gr5 S1	5	5	CF
DG_05-05-1	Objectif_10	0	75	20999,09	6987,25	0,33	17777	rat5 gr5 S1	5	5	DG
CPu_05-05-1	Objectif_10	0	113	91849,91	18581,84	0,2	47276	rat5 gr5 S1	5	5	Cpu
LGP_05-05-1	Objectif_10	0	108	40903,15	4619,13	0,11	11752	rat5 gr5 S1	5	5	LGP
CCx_05-05-1	Objectif_10	0	124	46707,71	7444,37	0,16	18940	rat5 gr5 S1	5	5	CCx
CF_06-05-1	Objectif_10	0	133	94868,92	10059,72	0,11	25594	rat6 gr5 S1	6	5	CF
DG_06-05-1	Objectif_10	0	96	14972,06	3380,23	0,23	8600	rat6 gr5 S1	6	5	DG
Cpu_06-05-1	Objectif_10	0	141	72270,12	7132,29	0,1	18146	rat6 gr5 S1	6	5	Cpu
LGP_06-05-1	Objectif_10	0	114	87078,67	17658,17	0,2	44926	rat6 gr5 S1	6	5	LGP
CCx_06-05-1	Objectif_10	0	91	45435,41	14336,89	0,32	36476	rat6 gr5 S1	6	5	CCx
CF_07-05-1	Objectif_10	0	141	67288,21	8531,55	0,13	21706	rat7 gr5 S1	7	5	CF
DG_07-05-1	Objectif_10	0	89	15344,67	4274,03	0,28	10874	rat7 gr5 S1	7	5	DG
CPu_07-05-1	Objectif_10	0	138	68753,5	5406,8	0,08	13756	rat7 gr5 S1	7	5	Cpu
LGP_07-05-1	Objectif_10	0	117	68972,03	12384,22	0,18	31508	rat7 gr5 S1	7	5	LGP
CCx_07-05-1	Objectif_10	0	106	62031,16	22589,77	0,36	57473	rat7 gr5 S1	7	5	CCx
CF_08-05-1	Objectif_10	0	129	84295,09	13340,51	0,16	33941	rat8 gr5 S1	8	5	CF
DG_08-05-1	Objectif_10	0	86	17501,34	4073,57	0,23	10364	rat8 gr5 S1	8	5	DG
CPu_08-05-1	Objectif_10	0	123	133475,9	17084,71	0,13	43467	rat8 gr5 S1	8	5	Cpu
LGP_08-05-1	Objectif_10	0	120	32202,59	8581,07	0,27	21832	rat8 gr5 S1	8	5	LGP
CCx_08-05-1	Objectif_10	0	25	30412,25	4425,74	0,15	11260	rat8 gr5 S1	8	5	CCx
CF_09-05-1	Objectif_10	0	129	110553,2	21030,93	0,19	53507	rat9 gr5 S1	9	5	CF
DG_09-05-1	Objectif_10	0	58	18288,23	4755,12	0,26	12098	rat9 gr5 S1	9	5	DG
CPu_09-05-1	Objectif_10	0	145	66831,48	5724,38	0,09	14564	rat9 gr5 S1	9	5	Cpu
CCx_09-05-1	Objectif_10	0	86	81343,68	33630,15	0,41	85562	rat9 gr5 S1	9	5	CCx
CF_10-05-1	Objectif_10	0	115	111827,5	13542,15	0,12	34454	rat10 gr5 S1	10	5	CF
DG_10-05-1	Objectif_10	0	84	26539,92	8082,29	0,3	20563	rat10 gr5 S1	10	5	DG
CPu_10-05-1	Objectif_10	0	139	101452,9	16671,22	0,16	42415	rat10 gr5 S1	10	5	Cpu
LGP_10-05-1	Objectif_10	0	93	64864,27	17931,34	0,28	45621	rat10 gr5 S1	10	5	LGP
CCx_10-05-1	Objectif_10	0	92	118897,3	40557,66	0,34	103187	rat10 gr5 S1	10	5	CCx
CF_11-05-1	Objectif_10	0	179	72240,25	10097,85	0,14	25691	rat11 gr5 S1	11	5	CF
DG_11-05-1	Objectif_10	0	107	16854,77	4948,5	0,29	12590	rat11 gr5 S1	11	5	DG
CPu_11-05-1	Objectif_10	0	160	43507,11	5109,65	0,12	13000	rat11 gr5 S1	11	5	Cpu
LGP_11-05-1	Objectif_10	0	128	61940,37	13588,92	0,22	34573	rat11 gr5 S1	11	5	LGP

CCx_11-05-1	Objectif_10	0	127	46662,51	17797,31	0,38	45280	rat11 gr5 S1	11	5	CCx
CF_12-05-1	Objectif_10	0	149	106718,6	15145,4	0,14	38533	rat12 gr5 S1	12	5	CF
DG_12-05-1	Objectif_10	0	114	25722,77	6479,82	0,25	16486	rat12 gr5 S1	12	5	DG
CPu_12-05-1	Objectif_10	0	172	40966,04	4077,5	0,1	10374	rat12 gr5 S1	12	5	Cpu
LGP_12-05-1	Objectif_10	0	121	47504,82	9008,31	0,19	22919	rat12 gr5 S1	12	5	LGP
CCx_12-05-1	Objectif_10	0	152	67660,03	14229,2	0,21	36202	rat12 gr5 S1	12	5	CCx
CF_13-05-1	Objectif_10	0	123	91351,13	14028,74	0,15	35692	rat13 gr5 S1	13	5	CF
DG_13-05-1	Objectif_10	0	88	13295,71	3720,22	0,28	9465	rat13 gr5 S1	13	5	DG
CPu_13-05-1	Objectif_10	0	141	84136,3	10088,02	0,12	25666	rat13 gr5 S1	13	5	Cpu
LGP_13-05-1	Objectif_10	0	129	75557,59	12535,94	0,17	31894	rat13 gr5 S1	13	5	LGP
CCx_13-05-1	Objectif_10	0	88	71290,64	22789,44	0,32	57981	rat13 gr5 S1	13	5	CCx
CF_14-05-1	Objectif_10	0	176	65445,98	6461,35	0,1	16439	rat14 gr5 S1	14	5	CF
DG_14-05-1	Objectif_10	0	103	20765,23	5314,43	0,26	13521	rat14 gr5 S1	14	5	DG
CPu_14-05-1	Objectif_10	0	151	26405,5	1239,68	0,05	3154	rat14 gr5 S1	14	5	Cpu
LGP_14-05-1	Objectif_10	0	141	36265,95	3270,57	0,09	8321	rat14 gr5 S1	14	5	LGP
CCx_14-05-1	Objectif_10	0	119	67236,72	20506,6	0,3	52173	rat14 gr5 S1	14	5	CCx
CF_15-05-1	Objectif_10	0	151	64502,66	5048,34	0,08	12844	rat15 gr5 S1	15	5	CF
DG_15-05-1	Objectif_10	0	142	10367,48	3080,73	0,3	7838	rat15 gr5 S1	15	5	DG
CPu_15-05-1	Objectif_10	0	160	33814,88	2217,98	0,07	5643	rat15 gr5 S1	15	5	Cpu
LGP_15-05-1	Objectif_10	0	144	56453,39	9339,26	0,17	23761	rat15 gr5 S1	15	5	LGP
CCx_15-05-1	Objectif_10	0	114	45079,3	9695,37	0,22	24667	rat15 gr5 S1	15	5	CCx
CF_16-05-1	Objectif_10	0	139	131069,2	18615,64	0,14	47362	rat16 gr5 S1	16	5	CF
DG_16-05-1	Objectif_10	0	112	20158,75	5227,57	0,26	13300	rat16 gr5 S1	16	5	DG
CCx_16-05-1	Objectif_10	0	84	62044,13	18471	0,3	46994	rat16 gr5 S1	16	5	CCx
CF_17-05-1	Objectif_10	0	189	94791,1	19639,14	0,21	49966	rat17 gr5 S1	17	5	CF
DG_17-05-1	Objectif_10	0	145	14386,03	3776,43	0,26	9608	rat17 gr5 S1	17	5	DG
CPu_17-05-1	Objectif_10	0	183	64654,77	11359,93	0,18	28902	rat17 gr5 S1	17	5	Cpu
LGP_17-05-1	Objectif_10	0	137	66734,8	13876,24	0,21	35304	rat17 gr5 S1	17	5	LGP
CCx_17-05-1	Objectif_10	0	114	73264,93	21618,15	0,3	55001	rat17 gr5 S1	17	5	CCx
CF_18-05-1	Objectif_10	0	133	125557,5	19590,4	0,16	49842	rat18 gr5 S1	18	5	CF
DG_18-05-1	Objectif_10	0	107	34882,02	8048,49	0,23	20477	rat18 gr5 S1	18	5	DG
CPu_18-05-1	Objectif_10	0	151	103117,1	12662,11	0,12	32215	rat18 gr5 S1	18	5	Cpu
LGP_18-05-1	Objectif_10	0	110	58352,61	12047,38	0,21	30651	rat18 gr5 S1	18	5	LGP
CCx_18-05-1	Objectif_10	0	89	73861,97	27230,12	0,37	69279	rat18 gr5 S1	18	5	CCx
CF_19-05-1	Objectif_10	0	167	122199,7	23213,54	0,19	59060	rat19 gr5 S1	19	5	CF
DG_19-05-1	Objectif_10	0	109	24140,35	4891,9	0,2	12446	rat19 gr5 S1	19	5	DG
CPu_19-05-1	Objectif_10	0	140	58974,02	5231,5	0,09	13310	rat19 gr5 S1	19	5	Cpu
LGP_19-05-1	Objectif_10	0	125	38905,67	9819,18	0,25	24982	rat19 gr5 S1	19	5	LGP
CCx_19-05-1	Objectif_10	0	138	62203,32	26081,63	0,42	66357	rat19 gr5 S1	19	5	CCx
