# Supplementary material for: Repeated exposure to nanosecond high power pulsed microwaves increases cancer incidence in rat
Source: PLoS One. 2020 Apr 8;15(4):e0226858. doi: 10.1371/journal.pone.0226858 (PMC7141660; doi:10.1371/journal.pone.0226858)
Supplement: S4 Table — Magnification x10. (PDF) [file pone.0226858.s005.pdf]

Nom de l'image	Calibration	DO min	DO max	Surface	Surface marquée	Ratio	Nombre
CF_E1-2	Objectif_10	0	107	143052,1	56232,1	0,39	143066
DG_E1-2	Objectif_10	0	73	19776,31	6421,26	0,32	16337
CPu_E1-2	Objectif_10	0	91	103113,5	29795,55	0,29	75806
LGP_E1-2	Objectif_10	0	72	77320,42	29191,04	0,38	74268
CCx_E1-2	Objectif_10	0	61	38178,53	14825,46	0,39	37719
CF_E2-2	Objectif_10	0	97	123131,2	37734,38	0,31	96004
DG_E2-2	Objectif_10	0	65	24254,33	8155,4	0,34	20749
CPu_E2-2	Objectif_10	0	87	74360,75	21551,72	0,29	54832
LGP_E2-2	Objectif_10	0	68	65133,9	18718,22	0,29	47623
CCx_E2-2	Objectif_10	0	54	49859,19	15462,2	0,31	39339
CF_E3-2	Objectif_10	0	76	105068,6	17223,85	0,16	43821
DG_E3-2	Objectif_10	0	53	21344,19	6364,27	0,3	16192
CPu_E3-2	Objectif_10	0	75	88466,14	21101,68	0,24	53687
LGP_E3-2	Objectif_10	0	55	42153,84	12867,28	0,31	32737
CCx_E3-2	Objectif_10	0	47	58616,74	17955,71	0,31	45683
DG_E4-2	Objectif_10	0	62	19939,43	4993,7	0,25	12705
CPu_E4-2	Objectif_10	0	83	67072,82	18000,51	0,27	45797
LGP_E4-2	Objectif_10	0	55	55054,52	15569,89	0,28	39613
CF_E5-2	Objectif_10	0	81	119897,2	26710,9	0,22	67958
DG_E5-2	Objectif_10	0	48	19989,74	4639,96	0,23	11805
CPu_E5-2	Objectif_10	0	74	85160,98	15512,11	0,18	39466
LGP_E5-2	Objectif_10	0	64	42515,05	15294,76	0,36	38913
CCx_E5-2	Objectif_10	0	46	71684,08	22407,39	0,31	57009
CF_E6-2	Objectif_10	0	82	118336	22644,4	0,19	57612
DG_E6-2	Objectif_10	0	57	10266,86	6286,44	0,61	15994
CPu_E6-2	Objectif_10	0	76	83520	17444,35	0,21	44382
LGP_E6-2	Objectif_10	0	55	59685,44	18190,36	0,3	46280
CCx_E6-2	Objectif_10	0	61	55842,2	18065,76	0,32	45963
CF_E7-2	Objectif_10	0	74	136454	33562,94	0,25	85391
DG_E7-2	Objectif_10	0	52	21787,16	7261,99	0,33	18476
CPu_E7-2	Objectif_10	0	72	127887,1	33592,81	0,26	85467
LGP_E7-2	Objectif_10	0	57	55144,93	16409,05	0,3	41748
CCx_E7-2	Objectif_10	0	47	35874,47	12883	0,36	32777
CF_E8-2	Objectif_10	0	76	114300,1	26813,88	0,23	68220
DG_E8-2	Objectif_10	0	62	32004,89	10865,48	0,34	27644
CPu_E8-2	Objectif_10	0	85	103738,9	27125,56	0,26	69013
LGP_E8-2	Objectif_10	0	83	27322,48	9582,95	0,35	24381
CCx_E8-2	Objectif_10	0	60	49180,39	20993,98	0,43	53413
CF_E9-2	Objectif_10	0	78	163681,4	46799,29	0,29	119067
DG_E9-2	Objectif_10	0	68	17123,23	4690,66	0,27	11934
CPU_E9-2	Objectif_10	0	81	142009,4	34090,8	0,24	86734
LGP_E9-2	Objectif_10	0	64	57280,37	18947,37	0,33	48206
CCx_E9-2	Objectif_10	0	46	110648,3	32661,68	0,3	83098
CF_E10-2	Objectif_10	0	62	147401,6	54392,23	0,37	138385
DG_E10-2	Objectif_10	0	50	16853,99	3154,62	0,19	8026
CPu_E10-2	Objectif_10	0	54	109276,6	21886,21	0,2	55683
LGP_E10-2	Objectif_10	0	47	51847,23	18351,12	0,35	46689
CCx_E10-2	Objectif_10	0	45	48788,91	21281,7	0,44	54145
CF_E11-2	Objectif_10	0	55	111425,4	25166,6	0,23	64029
DG_E11-2	Objectif_10	0	40	16526,97	3684,45	0,22	9374
CPu_E11-2	Objectif_10	0	64	131205,6	55657,86	0,42	141605
LGP_E11-2	Objectif_10	0	62	45644,91	18200,97	0,4	46307
CCx_E11-2	Objectif_10	0	51	45455,06	18036,68	0,4	45889
CF_E12-2	Objectif_10	0	67	152094,3	38491,39	0,25	97930

DG_E12-2	Objectif_10	0	42	25111,97	8424,64	0,34	21434
CPu_E12-2	Objectif_10	0	70	92222,13	24178,08	0,26	61514
LGP_E12-2	Objectif_10	0	62	54598,59	16396,87	0,3	41717
CCx_E12-2	Objectif_10	0	72	62118,42	16499,85	0,27	41979
CF_S1-2	Objectif_10	0	49	113329,3	18508,34	0,16	47089
DG_S1-2	Objectif_10	0	42	17798,88	3030,81	0,17	7711
CPu_S1-2	Objectif_10	0	43	123207	13734,74	0,11	34944
LGP_S1-2	Objectif_10	0	48	57265,82	12066,24	0,21	30699
CF_S2-2	Objectif_10	0	53	102350,2	19095,16	0,19	48582
CPu_S2-2	Objectif_10	0	58	103334	16506,14	0,16	41995
LGP_S2-2	Objectif_10	0	40	33255,97	6999,83	0,21	17809
CCx_S2-2	Objectif_10	0	34	53221,73	6624,07	0,12	16853
CF_S3-2	Objectif_10	0	49	79540,37	12779,63	0,16	32514
DG_S3-2	Objectif_10	0	39	27339,78	2749,78	0,1	6996
CPu_S3-2	Objectif_10	0	48	58097,52	5660,31	0,1	14401
LGP_S3-2	Objectif_10	0	47	36589,82	6712,12	0,18	17077
CCx_S3-2	Objectif_10	0	24	54404,81	7023,41	0,13	17869
CF_S4-2	Objectif_10	0	47	109707,7	6999,04	0,06	17807
DG_S4-2	Objectif_10	0	38	20518,39	4154,54	0,2	10570
CPu_S4-2	Objectif_10	0	55	76303,2	6732,95	0,09	17130
LGP_S4-2	Objectif_10	0	38	52533,5	5293,99	0,1	13469
CCx_S4-2	Objectif_10	0	30	42605,45	5720,06	0,13	14553
CF_S5-2	Objectif_10	0	59	107513,7	7097,31	0,07	18057
DG_S5-2	Objectif_10	0	42	21646,84	3890,8	0,18	9899
CPu_S5-2	Objectif_10	0	54	90240,76	12900,3	0,14	32821
LGP_S5-2	Objectif_10	0	42	31884,22	5430,38	0,17	13816
CCx_S5-2	Objectif_10	0	34	30332,07	3189,6	0,11	8115
DG_S6-2	Objectif_10	0	36	19495,28	1829,65	0,09	4655
CPu_S6-2	Objectif_10	0	57	96726,48	11842,99	0,12	30131
LGP_S6-2	Objectif_10	0	42	39971,23	8419,92	0,21	21422
CCx_S6-2	Objectif_10	0	38	36666,07	5424,09	0,15	13800
DG_S7-2	Objectif_10	0	47	23833,77	3855,43	0,16	9809
CPu_S7-2	Objectif_10	0	56	99861,84	8370,79	0,08	21297
LGP_S7-2	Objectif_10	0	39	55496,71	10052,65	0,18	25576
CF_S8-2	Objectif_10	0	83	66996,17	6032,53	0,09	15348
DG_S8-2	Objectif_10	0	43	30155,2	3961,95	0,13	10080
CPu_S8-2	Objectif_10	0	63	118383,5	13641,59	0,12	34707
LGP_S8-2	Objectif_10	0	54	37516,24	7420	0,2	18878
CCx_S8-2	Objectif_10	0	41	43632,49	7536,34	0,17	19174
CF_S9-2	Objectif_10	0	76	117739,7	11158,3	0,09	28389
DG_S9-2	Objectif_10	0	46	20302,61	3185,67	0,16	8105
CPu_S9-2	Objectif_10	0	81	55504,18	13153,03	0,24	33464
LGP_S9-2	Objectif_10	0	57	49532,17	7506,86	0,15	19099
CF_S10-2	Objectif_10	0	68	88692,14	9241,39	0,1	23512
DG_S10-2	Objectif_10	0	47	15056,57	1936,56	0,13	4927
CPu_S10-2	Objectif_10	0	58	86445,86	4253,98	0,05	10823
LGP_S10-2	Objectif_10	0	45	32101,97	5727,53	0,18	14572
CCx_S10-2	Objectif_10	0	61	55425,56	8832,62	0,16	22472
CF_S11-2	Objectif_10	0	72	74758,91	6705,04	0,09	17059
DG_S11-2	Objectif_10	0	50	16940,85	2461,67	0,15	6263
CPu_S11-2	Objectif_10	0	64	50932,21	3766,21	0,07	9582
LGP_S11-2	Objectif_10	0	49	43997,24	8314,98	0,19	21155
CCx_S11-2	Objectif_10	0	55	30372,55	6758,5	0,22	17195
DG_S12-2	Objectif_10	0	93	24806,57	3580,69	0,14	9110
CPu_S12-2	Objectif_10	0	96	123949,5	15595,05	0,13	39677
LGP_S12-2	Objectif_10	0	79	63740,93	13762,26	0,22	35014

CCx_S12-2	Objectif_10	0	83	26139,4	5914,23	0,23	15047
