# Supplementary material for: Repeated exposure to nanosecond high power pulsed microwaves increases cancer incidence in rat
Source: PLoS One. 2020 Apr 8;15(4):e0226858. doi: 10.1371/journal.pone.0226858 (PMC7141660; doi:10.1371/journal.pone.0226858)
Supplement: S5 Table — (PDF) [file pone.0226858.s006.pdf]

	Benine		Malignant		Benine		Malignant	
	Number/Total				%			
	Ex	Sham	Ex	Sh	Ex	Sham	Ex	Sham
brain	1/24	-	-	-	4	-		
pituitary	5/24	4/23	1/24	-	21	17	4	-
thyroid/ parathyroid	-	-	-	-				
trachea/ pharynx	-	-	-	-				
lung	-	-	-	-				
heart	-	-	-	-				
intestine	-	-	1/12	1/23			8	4
lever	-	-	-	2/23			-	9
pancreas	1/6	3/23	1/24	-	17	13	4	-
spleen	-	-	-	-				
Lymph node/ thymus/blood	-	-	1/6	-			17	-
adrenals	5/24	5/23	1/12	-	21	22	8	-
kidney	-	-	1/24	-			4	-
bladder	-	-	-	-				
testes	1/24	-	-	-	4	-		
prostata	-	-	-	-				
preputial glands	-	-	-	1/23			-	4
bone	-	1/23	1/24	-	-	4	4	-
eyes/ears	1/24	-	-	-	4	-		
skin/sub-cutaneous	3/8	6/23	1/8	3/23	38	26	13	13
breast	1/12	-	1/24	-	8	-	4	-
